# Tumor copy number instability is a significant predictor for late recurrence after radical surgery of pancreatic ductal adenocarcinoma

**DOI:** 10.1002/cam4.3425

**Published:** 2020-08-30

**Authors:** Chenlei Wen, XiaXing Deng, Dandan Ren, Xue Song, Hao Chen, Jiancheng Wang, Jiabin Jin, Dongfeng Cheng, Zhiwei Xu, Jun Zhang, Junjie Xie, Wenjing Qi, Jiangning Gu, Chenghong Peng, Dan Chen, Shi Chen, Baiyong Shen, Qian Zhan

**Affiliations:** ^1^ Department of General Surgery Pancreatic Disease Center Research Institute of Pancreatic Diseases Ruijin Hospital Shanghai Jiao Tong University School of Medicine Shanghai China; ^2^ State Key Laboratory of Oncogenes and Related Genes Institute of Translational Medicine Shanghai Jiao Tong University Shanghai China; ^3^ Genecast Biotechnology Co., Ltd Wuxi China; ^4^ Department of Pathology The First affiliated Hospital of Dalian Medical University Dalian China; ^5^ Department of Hepatobiliary Surgery The First affiliated Hospital of Dalian Medical University Dalian China; ^6^ Department of Hepato‐Biliary‐Pancreatic Surgery Fujian Provincial Hospital Shengli Clinical Medical College of Fujian Medical University Fujian Medical University Fuzhou China

**Keywords:** copy number instability, late recurrence, pancreatic ductal adenocarcinoma, predictive marker, prognosis

## Abstract

**Objective:**

Our study examined the association between molecular features and clinical results of pancreatic ductal adenocarcinoma (PDAC) patients, aiming to explore the genomic determinants of the recurrence and prognosis of PDAC after surgical removal.

**Methods:**

This retrospective study analyzed 181 PDAC patients who received pancreatectomy and adjuvant chemotherapy, with 67 patients in the training set. An internal validation set of 48 patients and an external validation set of 66 patients were used to validate the result. Comprehensive genomic profiling was performed on formalin‐fixed paraffin‐embedded (FFPE) tumor specimens to determine genomic features using the designed cancer‐related gene panel based on next‐generation sequencing (NGS).

**Results:**

Significant differences were identified between the late recurrence (LR) group and early recurrence (ER) group in tumor copy number instability (CNI) levels. Next, the utility of low CNI (the middle and lowest tertile) with regard to predicting LR was confirmed in the training, internal, and external validation sets. Further univariate and multivariate analyses revealed that CNI was an independent predictive and prognostic biomarker, and had higher predictive accuracy for LR than CA19‐9 level, pathological stage, tumor size, and age. In addition, CNI combined with lymph node (LN) metastasis status could provide a more accurate model for predicting LR of PDAC.

**Conclusion:**

We discovered and validated the association between CNI and clinical outcome in 181 patients with resectable PDAC, demonstrating the utility of lower tumor CNI levels as biomarkers of postoperative LR and favorable prognosis. Moreover, the combination of CNI and LN metastasis status elevated the predictive accuracy and illuminated strategies for patient stratification.

## Graphical abstract

We discovered and validated the association between copy number instability (CNI) and clinical outcome in 181 patients with resectable pancreatic ductal adenocarcinoma (PDAC), demonstrating the utility of lower tumor CNI levels as biomarkers of postoperative late recurrence and favorable prognosis. Moreover, the combination of CNI and lymph node metastasis status elevated the predictive accuracy and illuminated strategies for patient stratification.

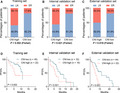

## 1 INTRODUCTION

Pancreatic cancer is one of the most aggressive human malignancies; it carries a poor prognosis, with a 5‐year survival rate of 8%, and it ranks fourth among all causes of cancer‐related death.^1^, ^2^ In China, pancreatic cancer is now the ninth leading cause of cancer‐related mortality, with a sharply increasing incidence rate.^3^ Pancreatic ductal adenocarcinoma (PDAC) is the most common type and accounts for more than 90% of pancreatic cancer.^4^ Despite considerable improvements in multiline therapeutic management, surgical resection still remains the only potentially curative treatment for patients with PDAC.^5^ Furthermore, adjuvant chemotherapy with gemcitabine or S‐1 and other conservative therapies are necessary to palliate symptoms and improve survival in patients with resectable PDAC.^6^, ^7^, ^8^

High incidences of local recurrence and distant metastasis are two of the main reasons for the poor outcome of PDAC.^9^ To date, several postoperative features have been reported as predictive and prognostic markers in PDAC. Postoperative features, such as lymph node (LN) metastasis, tumor size (>3 cm), tumor grade, resection margin, and differentiation, have been extensively developed as predictors of survival and recurrence.^6^, ^10^, ^11^, ^12^, ^13^ Serum carbohydrate antigen 19‐9 (CA19‐9) is a well‐known preoperative and postoperative predictor of clinical outcomes in PDAC.^14^, ^15^, ^16^, ^17^ However, the evaluation of patient prognosis mainly relies on pathological features and serum tumor markers. In addition to the four major driver genes (*KRAS*, *CDKN2A*, *TP53*, and *SMAD4*), few established genomic features have been reported as postoperative predictive markers of outcomes in PDAC.^18^ Therefore, it is of great interest to further investigate the molecular features to identify more potential biomarkers to predict postoperative recurrence and survival in patients with resectable PDAC.

Next‐generation sequencing (NGS)‐based gene panel testing is a clinically useful tool for individualized cancer patient care that investigates genomic features related to targetable gene alterations, tumor mutational burden (TMB), and copy number instability (CNI). To explore the genomic determinants of the recurrence of PDAC after surgical removal, we retrospectively investigated the clinical characteristic and molecular features of 181 PDAC patients, with 67 patients in the training set, 48 patients in the internal, and 66 in the external validation set. The predictive and prognostic abilities of these genomic signatures, as well as clinical and laboratory factors, were extensively investigated. Our investigation helps identify PDAC patients who may not achieve a sustained therapeutic benefit after tumor resection, and for whom further treatment options should be considered.

## 2 MATERIALS AND METHODS

### 2.1 Study design and patients

Formalin‐fixed paraffin‐embedded (FFPE) tumor specimens and matched blood samples were collected from 220 patients who were diagnosed with PDAC. All the patients had undergone pancreatectomy and received first‐line adjuvant chemotherapy for at least 6 months. About 150 patients diagnosed at Ruijin Hospital, School of Medicine, Shanghai Jiao Tong University were randomly assigned (1:1) to a training set and an internal validation set, and their samples were profiled by a targeted NGS panel A. Seventy patients diagnosed at the First Affiliated Hospital, Dalian Medical University or Fujian provincial hospital, Shengli Clinical Medical College of Fujian Medical University were regarded as an external validation set and their samples were sequenced by a targeted NGS panel B.

Samples were excluded if they failed to meet the inclusion criteria (clinical quality control): lacking serum tumor marker levels or recurrence status, receiving neoadjuvant chemotherapy, or dying from other causes. All patients signed informed consent forms giving their consent to participate in the study prior to their enrollment. All experiments were carried out in accordance with relevant guidelines and regulations.

### 2.2 Follow‐up and definition of recurrence

For the first 3 years after pancreatectomy, the follow‐up interval was 3 months and comprised a physical examination, laboratory tests, measurement of tumor markers, and computed tomography (CT). Confirmed recurrence was defined as recurrence suspected on abdominal CT, which was confirmed as recurrence on subsequent follow‐up. Progression‐free survival (PFS) was calculated from the date of pancreatectomy to the date of recurrence or last follow‐up if recurrence did not occur. Patients who relapsed within 6 months after pancreatectomy were defined as experiencing early recurrence (ER), and patients who relapsed after 6 months or without recurrence were defined as experiencing late recurrence (LR).^14^, ^19^

### 2.3 Identification of somatic mutations

FFPE tumors and matched blood samples were submitted for NGS. The NimbleDesign assay was used to identify mutations. We used VarScan2 with the following filters: (a) located in intergenic regions or intronic regions; (b) synonymous single‐nucleotide variants (SNVs); (c) allele frequency ≥0.005 in the Exome Aggregation Consortium (ExAC) database; (d) allele frequency <0.01 in the tumor sample; and (e) support reads <5. To identify somatic SNVs and indel mutations, matched blood samples from patients were used as controls for the FFPE tumor samples with mutations.

### 2.4 Calculation of TMB

To calculate the TMB value of the FFPE tumor samples, the number of somatic nonsynonymous SNVs detected by NGS was quantified by excluding (a) alterations with allele frequency ≥0.002 in the ExAC database; (b) those recorded in the COSMIC database; and (c) those with variant allele frequencies (VAFs) between 0.01 and 1. The TMB was measured in mutations per Mb.

### 2.5 CNI evaluation

We used the “‐normal” parameter to construct the copy number baseline as a negative control, and used a copy number variant (CNV) kit to call copy number variations from the FFPE tumor samples for each patient. For the determination of the FFPE tumor sample CNI, the copy numbers were called after mapping using the Burrows‐Wheeler Alignment (BWA) tool. After correction for GC content and mappability using proprietary algorithms for tumor DNA sequencing, the read counts were transformed into log2 ratios and converted into *Z*‐values based on Gaussian transformations vs a normal control group. The target regions that satisfied the *Z*‐score greater than the 95th percentile plus twice the absolute standard deviation of the normal control group were retained, and these *Z*‐scores were summed as the CNI.

### 2.6 Statistical analysis

The Fisher exact test was applied to explore the relationship between CNI and clinical and molecular characteristics, as well as the distribution of gene mutations in the ER and LR groups. The Mann‐Whitney test was used to assess the differences in CNI and TMB levels, which were regarded as continuous variables, between the ER and LR groups. Recurrence curves were obtained by Kaplan‐Meier analysis with the log‐rank test. Logistic regression analysis was performed to assess the factors predictive of LR, and those with *P* < .10 were selected for inclusion in the subsequent multivariate analysis. Receiver operating characteristic (ROC) curves were constructed to assess the ability of CNI to predict LR with the area under the curve (AUC) representing the predictive performance. Statistical analyses were carried out using R (https://www.r‐project.org/, version 3.6.2), and *P* < .05 was considered statistically significant.

## 3 RESULTS

### 3.1 Clinical characteristics of studied PDAC patients

Of the 220 patients, 39 were excluded after clinical data quality control. Finally, 181 PDAC patients were included in this study. Of the 115 enrolled patients from Ruijin hospital, 67 were in the training set and 48 were in the internal validation set. The other 66 patients from the First Affiliated Hospital and Fujian provincial hospital were regarded as an external validation set (Figure S1). As shown in Table 1, the median age was 61.0 years with interquartile range (IQR) from 52.0 to 65.0 years, 134 patients had early‐stage disease (stage I‐II), and 47 patients had advanced‐stage disease (stage III‐IV). According to the latest follow‐up, 55 patients had experienced ER after pancreatectomy, whereas 126 patients had experienced LR (42 patients relapsed after 6 months, and 84 patients had no recurrence).

**TABLE 1:** Clinical and laboratory characteristics of the studied PDAC patients

Characteristics	Whole cohort	Training set	Internal validation set	External validation set	*P* value
Total (cases)	181	67	48	66	
Gender (cases (%))					.699
Female	52 (28.7)	18 (26.9)	16 (33.3)	18 (27.3)	
Male	129 (71.3)	49 (73.1)	32 (66.7)	48 (72.7)	
Age (years)					.053
Median	61.0	58.0	59.0	62.5	
IQR	52.00‐65.00	51.50‐63.00	50.00‐65.25	54.25‐66.75	
Pathological stage (cases (%))					.227
Advanced	47 (26.0)	22 (32.8)	12 (25.0)	13 (19.7)	
Early	134 (74.0)	45 (67.2)	36 (75.0)	53 (80.3)	
T status (cases (%))					.386
T3/T4	70 (38.7)	26 (33.8)	15 (31.3)	29 (43.9)	
T1/T2	111 (61.3)	41 (66.2)	33 (68.7)	37 (56.1)	
LN metastasis status (cases (%))					.474
Positive	80 (44.2)	26 (38.8)	24 (50.0)	30 (45.5)	
Negative	101 (55.8)	41 (61.2)	24 (50.0)	36 (54.5)	
Tumor size (cm) (cases (%))					.343
>3	94 (51.9)	39 (58.2)	25 (52.1)	30 (45.5)	
≤3	87 (48.1)	28 (41.8)	23 (47.9)	36 (54.5)	
CA19‐9 (U/mL) (cases (%))					.180
Elevated (≥37)	130 (71.8)	44 (65.7)	39 (81.3)	47 (71.2)	
Normal (<37)	51 (28.2)	23 (34.3)	9 (18.7)	19 (28.8)	
Recurrence (cases (%))					.937
Early	55 (30.4)	21 (31.3)	15 (31.2)	19 (28.8)	
Late	126 (69.6)	46 (68.7)	33 (68.8)	47 (71.2)	

Note

*P* value: Kruskal‐Wallis test or Fisher's exact test (two sided) was used for the comparison between the training, internal, and external validation sets.

Abbreviations: CA19‐9, preoperative carbohydrate antigen 19‐9; CEA, preoperative carcinoembryonic antigen; IQR, Interquartile range; LN, lymph node.

### 3.2 Differential molecular features between ER and LR groups

**FIGURE 1:**
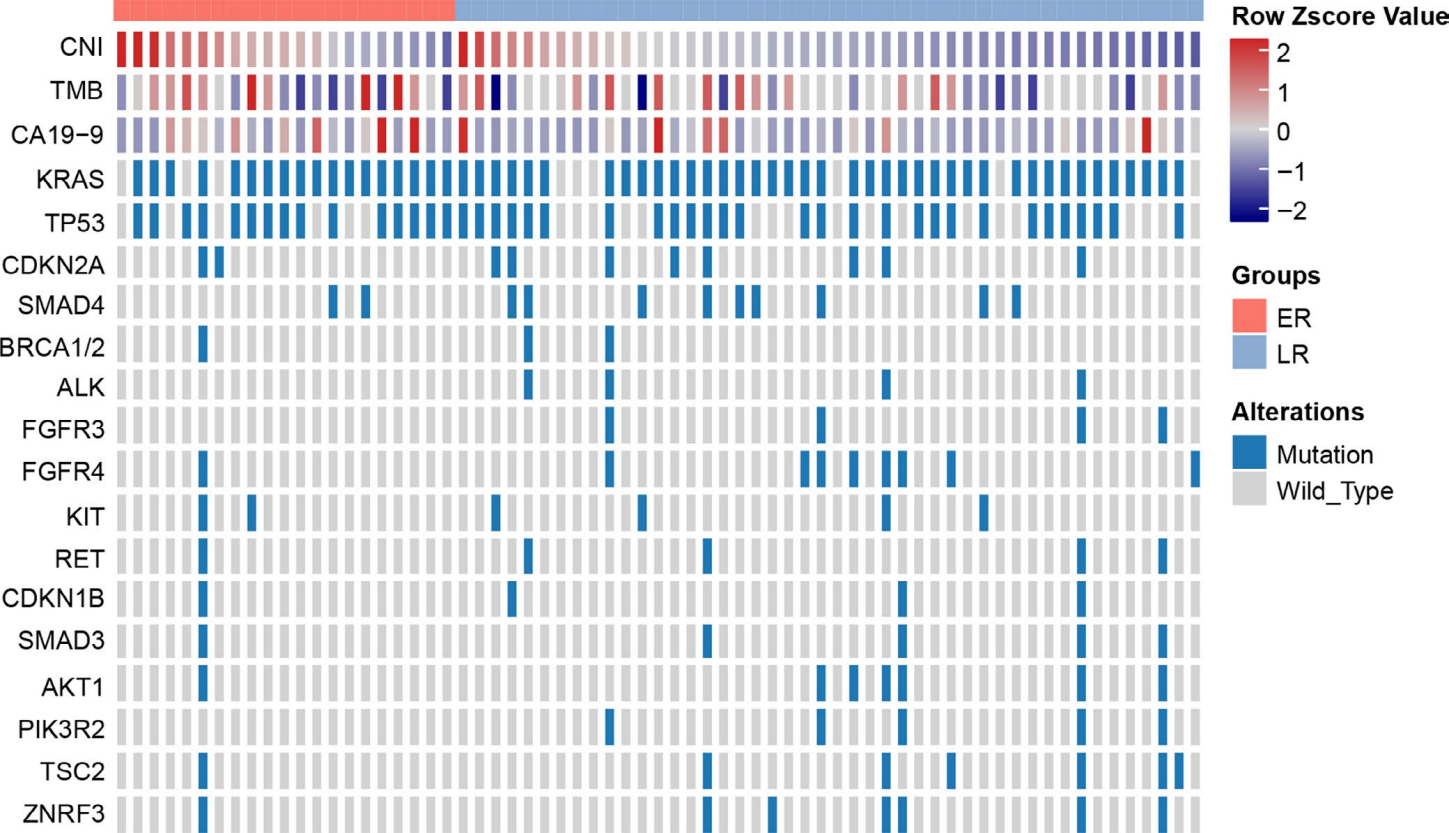
Differences in molecular features between ER and LR groups

To investigate the predictive markers for PDAC, molecular features were assessed in the current study. As demonstrated in Figure 1 and Table 2, in the ER and LR groups, of the four major driver genes, *KRAS* was mutated most frequently, with rates of 85.7% (18/21) and 87.0% (40/46), respectively. Moreover, *TP53* had mutation rates of 71.4% (15/21) and 60.9% (28/46), *CDKN2A* had mutation rates of 9.5% (2/21) and 17.4% (8/46), and *SMAD4* had mutation rates of 9.5% (2/21) and 19.6% (9/46) in the ER and LR groups, respectively. Unfortunately, there were no significant differences in the distributions of these gene mutations between the ER and LR groups (Fisher's exact test, *P* > .05).

**TABLE 2:** The clinical and molecular features between early and late recurrence groups in the training set

Characteristics	All patients	ER	LR	*P* value
Total (cases)	67	21	46	
Gender (cases (%))				.235
Female	18 (26.9)	8 (38.1)	10 (21.7)	
Male	49 (73.1)	13 (61.9)	36 (78.3)	
Age (years)				.730
Median	58	60	58	
IQR	51.50‐63.00	51.00‐63.00	52.00‐62.75	
Pathological stage (cases (%))				.271
Advanced	22 (32.8)	9 (42.9)	13 (28.3)	
Early	45 (67.2)	12 (57.1)	33 (71.7)	
T status (cases (%))				.058
T3/T4	26 (33.8)	12 (57.1)	14 (30.4)	
T1/T2	41 (66.2)	9 (42.9)	32 (69.6)	
LN metastasis status (cases (%))				.058
Positive	26 (38.8)	12 (57.1)	14 (30.4)	
Negative	41 (61.2)	9 (42.9)	32 (69.6)	
Tumor size (cm) (cases (%))				.185
>3	39 (58.2)	15 (71.4)	24 (52.2)	
≤3	28 (41.8)	6 (28.6)	22 (47.8)	
CA19‐9 (U/mL) (cases (%))				.587
Elevated (≥37)	44 (65.7)	15 (71.4)	29 (63.0)	
Normal (<37)	23 (34.3)	6 (28.6)	17 (37.0)	
CEA (ng/mL) (cases (%))				.553
Elevated (≥5)	18 (26.9)	7 (33.3)	11 (23.9)	
Normal (<5)	49 (73.1)	14 (66.7)	35 (76.1)	
CNI				.002
Median	5490.03	7616.53	5303.24	
IQR	4587.06‐7629.59	5414.15‐9484.42	4314.19‐6479.89	
TMB (Mutations/Mb)				.735
Median	5.99	6	5.99	
IQR	4.00‐7.97	3.99‐7.97	4.00‐7.97	
*KRAS* (cases (%))				1.000
Mutation	58 (86.6)	18 (85.7)	40 (87.0)	
Wild‐type	9 (13.4)	3 (14.3)	6 (13.0)	
*TP53* (cases (%))				.584
Mutation	43 (64.2)	15 (71.4)	28 (60.9)	
Wild‐type	24 (35.8)	6 (28.6)	18 (39.1)	
*CDKN2A* (cases (%))				.487
Mutation	10 (14.9)	2 (9.5)	8 (17.4)	
Wild‐type	57 (85.1)	19 (90.5)	38 (82.6)	
*SMAD4* (cases (%))				.481
Mutation	11 (16.4)	2 (9.5)	9 (19.6)	
Wild‐type	56 (83.6)	19 (90.5)	37 (80.4)	
*BRCA1/2* (cases (%))				1.000
Mutation	3 (4.5)	1 (4.8)	2 (4.3)	
Wild‐type	64 (95.5)	20 (95.2)	44 (95.7)	

Note

*P* value: Mann‐Whitney test rank sum or Fisher's exact test (two sided) was used for the comparison between the early and late recurrence groups.

Abbreviations: *BRCA1/2*, breast cancer 1/2; CA19‐9, preoperative carbohydrate antigen 19‐9; *CDKN2A*, cyclin‐dependent kinase inhibitor 2A; CEA, preoperative carcinoembryonic antigen; CNI, copy number instability; ER, early recurrence; IQR, Interquartile range; *KRAS*, v‐Ki‐ras2 Kirsten rat sarcoma viral oncogene homolog; LN, lymph node; LR, late recurrence; *SMAD4*, SMAD family member 4; TMB, tumor mutation burden; *TP53*, tumor protein p53.

In addition to gene alterations, the CNI and TMB were further calculated in our cohort. The results showed that the median CNI in the training set was 5490.03 (IQR from 4587.06 to 7629.59), and the median TMB value was 5.99 mutations/Mb (IQR from 4.00 to 7.97). As shown in Table 2, the CNI was significantly higher in the ER group than in the LR group (Mann‐Whitney test, *P* = .002). However, the TMB value was comparable between the ER and LR groups (Mann‐Whitney test, *P* = .735).

### 3.3 The predictive performance of CNI in PDAC

**FIGURE 2:**
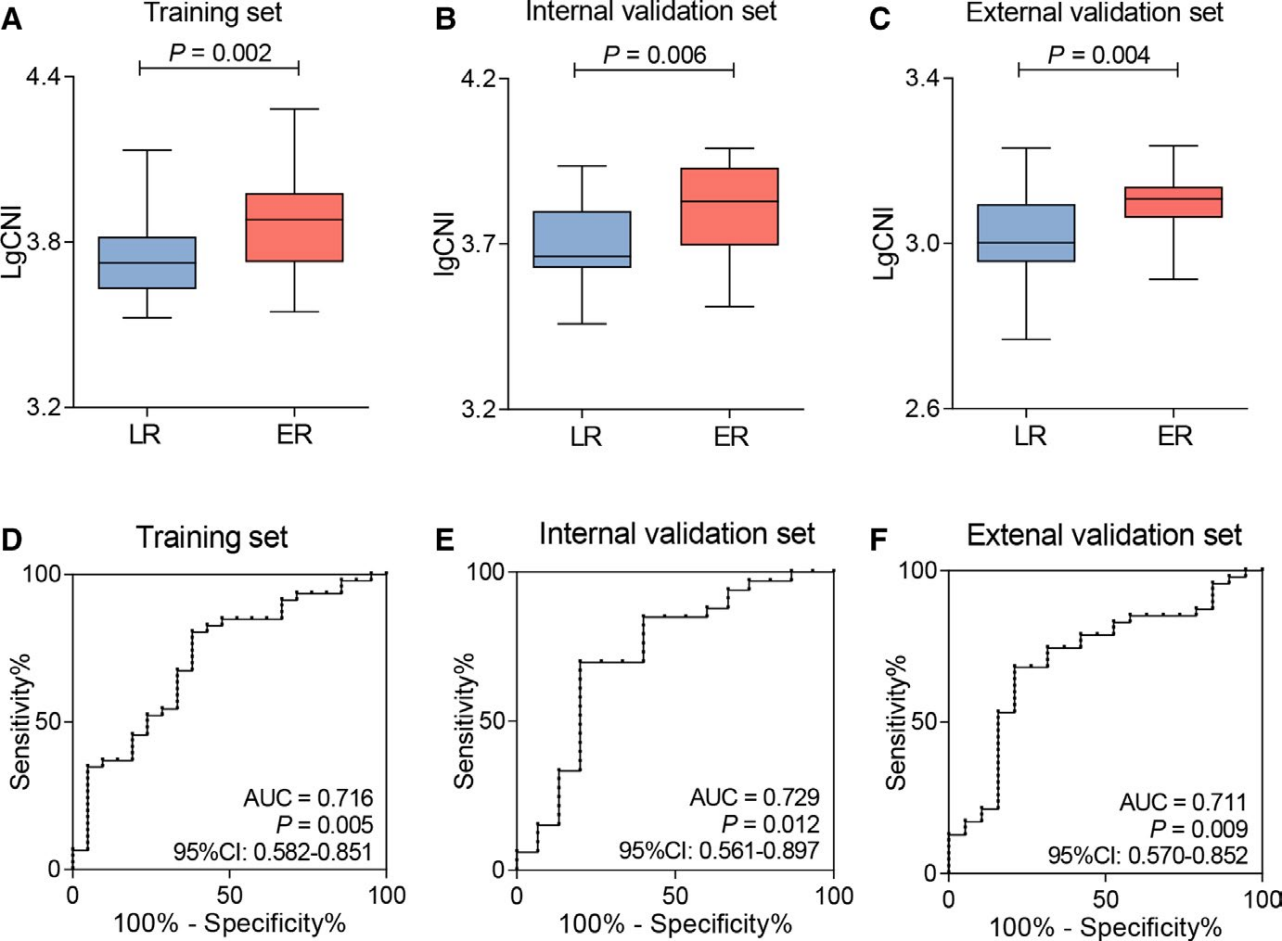
The predictive performance of CNI in PDAC. Dot‐box plots of CNI in ER and LR groups in (A) the training set, (B) the internal validation set, and (C) the external validation set. ROC curves for the ability of CNI to evaluate late recurrence in (D) the training set, (E) the internal validation set, and (F) the external validation set

Based on the significant difference in CNI between the ER and LR groups in the training set (Mann‐Whitney test, *P* = .002, Table 2 and Figure 2A), we next identified that CNI was also significantly lower in the LR group than in the ER group in both the internal and external validation sets (Mann‐Whitney test; internal validation set, *P* = .006; external validation set, *P* = .004; Figure 2B,C). Subsequently, the ROC analyses indicated a significant value of CNI for predicting LR in all three cohorts, with an AUC of 0.716 (training set, *P* = .005), 0.729 (internal validation set, *P* = .012), and 0.711 (external validation set, *P* = .009), respectively (Figure 2D‐F).

**FIGURE 3:**
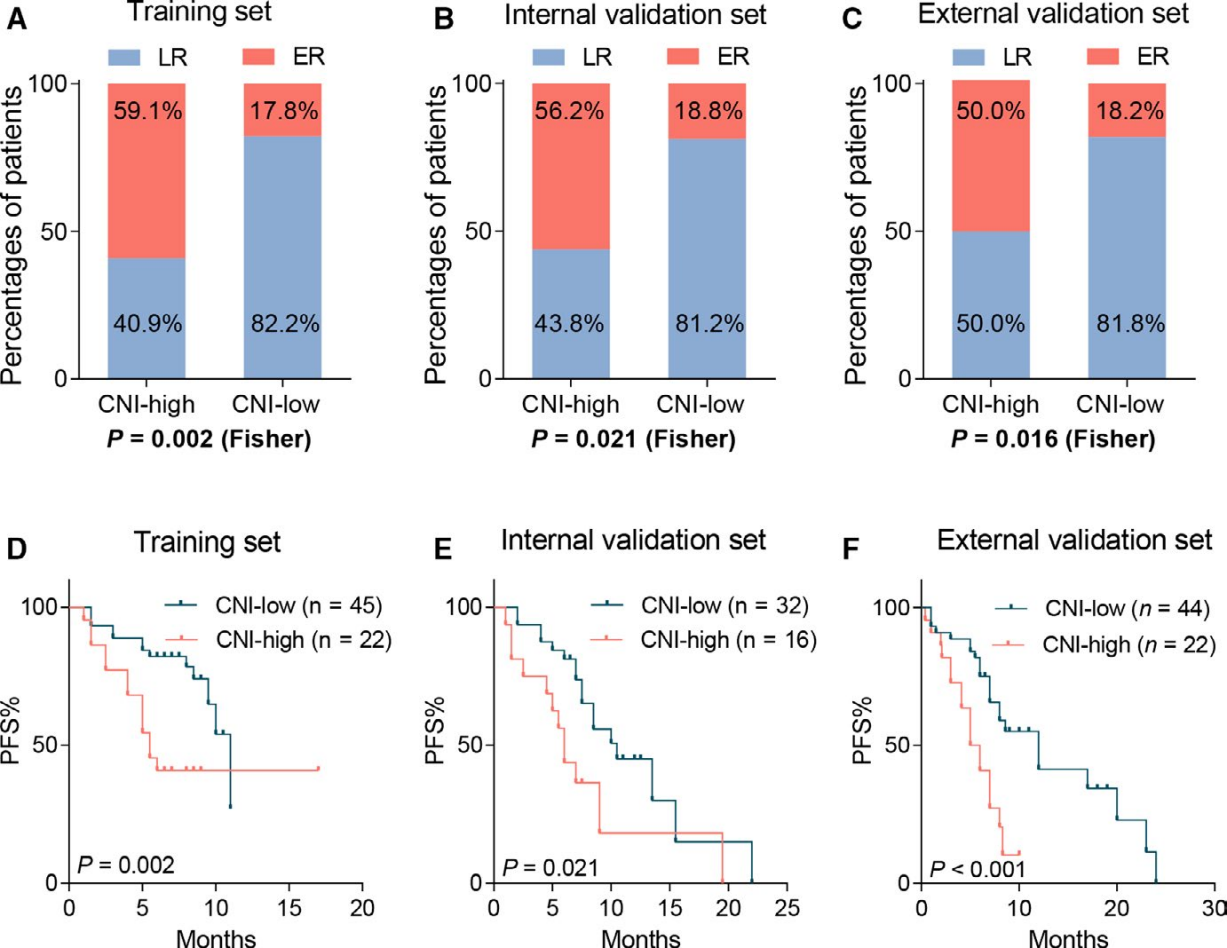
CNI was inversely correlated with clinical outcome in PDAC. Distribution of early recurrence and late recurrence in high or low CNI subgroups in (A) the training set, (B) the internal validation set, and (C) the external validation set. Kaplan‐Meier analyses for CNI above vs below the optimal threshold value for (D) the internal training set, (E) the validation set, and (F) the external validation set

To further investigate the ability of CNI to predict LR, we defined the CNI‐high group as that with a CNI of highest tertile and the CNI‐low group as that with a CNI of middle and lowest tertile. The LR rate was compared between the CNI‐high and CNI‐low groups. Surprisingly, the CNI‐low group had a very high rate of LR (Fisher's exact test, training set, 82.2%, *P* = .002, Figure 3A; internal validation set, 81.2%, *P* = .020, Figure 3B; external validation set, 81.8%, *P* = .016, Figure 3C). The above data indicated that a low CNI could be a predictive signature for LR in PDAC.

Clinical and laboratory features, such as LN metastasis, preoperative CA19‐9, pathological stage, tumor size, and age, have been reported to be associated with recurrence in PDAC.^6^, ^17^, ^20^, ^21^ Herein, univariate and multivariable logistic regression analyses were performed to assess whether the following characteristics were risk factors for LR: age, gender, pathological stage, T status, LN metastasis status, tumor size, preoperative CA19‐9, and preoperative carcinoembryonic antigen (CEA). Univariate analysis demonstrated T status, LN metastasis status, and CNI as considerable risk factors for LR (*P* < .050, Table 3). Further, multivariable analysis showed that both CNI and LN metastasis status were independent predictive factors for recurrence in PDAC patients (CNI: HR = 7.918, 95% CI, 2.128‐29.458, *P* = .002; LN metastasis status, HR = 4.230, 95% CI, 1.153‐15.518, *P* = .030), and that CNI might be a better predictive marker than LN metastasis status (Table 3).

**TABLE 3:** Univariate and multivariate analyses of predictive factors in the training set

Factors	Categories	Univariate analysis			Multivariate analysis		
		OR	95% CI	*P* value	OR	95% CI	*P* value
Age (years)	>58 vs ≤58	1.200	0.427‐3.373	.730			
Gender	Male vs female	0.451	0.146‐1.391	.166			
Pathological stage	Advanced vs early	1.904	0.649‐5.587	.241			
T status	T3/T4 vs T1/T2	3.048	1.047‐8.870	**.041**	2.028	0.597‐6.891	.257
LN metastasis status	Positive vs negative	3.048	1.047‐8.870	**.041**	4.230	1.153‐15.518	**.030**
Tumor size (cm)	>3.0 vs ≤3.0	2.292	0.756‐6.950	.143			
CA19‐9 (U/mL)	Elevated vs normal	1.466	0.478‐4.492	.504			
CEA (ng/mL)	Elevated vs normal	1.591	0.513‐4.936	.422			
CNI	High vs low	6.681	2.130‐20.950	**.001**	7.918	2.128‐29.458	**.002**

Note

*P* value: Wald test in logistic regression analysis, bold figures indicated statistically significance.

Abbreviations: CA19‐9, preoperative carbohydrate antigen 19‐9; CEA, preoperative carcinoembryonic antigen; CI, confidence interval; CNI, copy number instability; LN, lymph node; OR, Odds ratio; TMB, tumor mutation burden.

### 3.4 The prognostic value of CNI in PDAC

Given the predictive performance of CNI in PDAC, we further investigated the prognostic value of CNI in PDAC. K‐M plotter was used to compare PFS in the CNI‐high and CNI‐low groups (CNI‐high: highest tertile, CNI‐low: middle and lowest tertile). Of note, the CNI‐low group had a significantly longer PFS than the CNI‐high group in both the three cohorts (log‐rank test, training set, median PFS, 11.0 vs 5.5 months, HR = 0.368, *P* = .002, Figure 3D; internal validation set, median PFS, 10.5 vs 6.0 months, HR = 0.444; *P* = .021, Figure 3E; external validation set, median PFS, 12.0 vs 5.5 months, HR = 0.367, *P* < .001, Figure 3F). These results indicated that CNI was inversely related to prognosis in pancreatic cancers.

### 3.5 Joint utility of CNI with LN metastasis status

**FIGURE 4:**
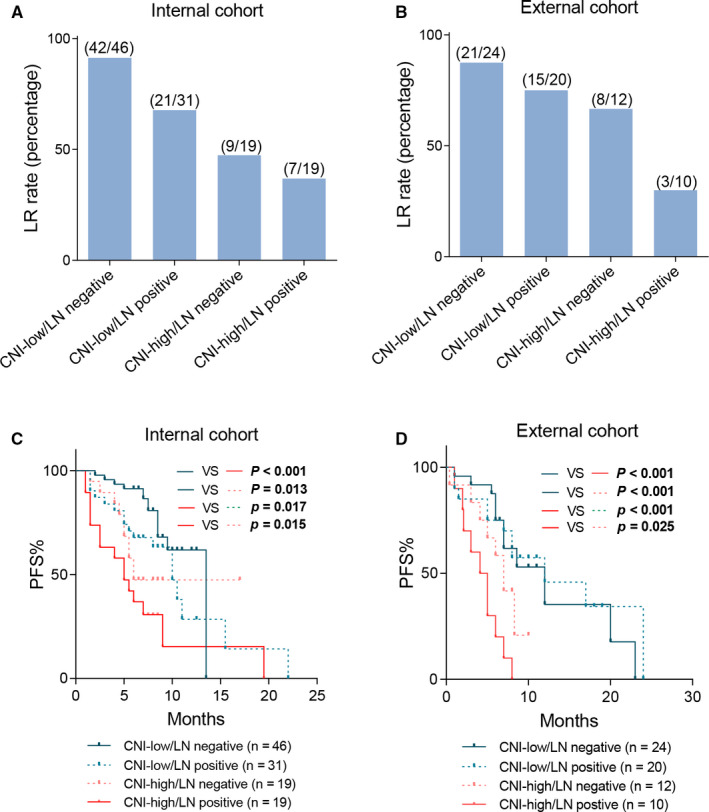
Combined utility of CNI with LN metastasis status. Distribution of early recurrence and late recurrence in four defined subgroups of (A) the internal cohort and (B) the external cohort. Kaplan‐Meier analysis for the combination of CNI and LN metastasis status in (C) the internal cohort and (D) the external cohort

Given the fact that CNI and LN metastasis status were independent predictive factors for LR in our study population (Table 3), we further explored the possibility of combining CNI with LN metastasis status for the prediction of LR in PDAC patients. Intriguingly, we observed that CNI‐low and LN‐negative subgroup had an extremely high LR rate of 91.3% (42/46) in the internal cohort (the training set and internal validation set, Figure 4A) and 87.5% (24/26) in the external cohort (the external validation set, Figure 4C), respectively. Moreover, patients within the CNI‐low/LN‐negative subgroup had a significantly longer PFS than the other three subgroups (log‐rank test for trend, internal cohort, *P* = .003, Figure 4B; external cohort, *P* < .001, Figure 4D). In all, the above data suggested that there is value in combining CNI and LN metastasis status when evaluating the prognosis of PDAC patients.

## 4 DISCUSSION

Despite considerable achievements in diagnosis and therapeutic strategies, the overall 5‐year survival rate of PDAC has remained dismal in the past few years.^22^, ^23^, ^24^ Therefore, more reproducible and utilizable biomarkers are needed to provide appropriate adjuvant treatment protocols for patients with resected PDAC. In the present study, we retrospectively evaluated the baseline genomic and clinical features of a PDAC cohort of patients who underwent pancreatectomy and adjuvant chemotherapy. Here, we show that the CNI level may have superior predictive value compared with other signatures, including CA19‐9, T status, LN metastasis status, SNV, and TMB.

Previously, four major driver gene alterations were found to be associated with poor prognosis in PDAC patients.^18^, ^25^, ^26^, ^27^, ^28^, ^29^ However, no significant differences in the distribution patterns of these genes were identified in our cohort, which may possibly be the result of the imbalanced proportions of patients between the mutation and wild‐type groups. A larger sample size may further support the significance of the driver gene signature. Moreover, previous studies have demonstrated the prognostic value of LN metastasis status.^21^ As expected, we herein identified that LN metastasis status was significantly associated with LR and was also independent predictive factor for recurrence in PDAC patients (Table 3). Furthermore, clinical and laboratory features (including CA19‐9 level, tumor size, and pathological stage), which are well‐known prognostic factors,^6^, ^11^, ^19^ were analyzed in our cohort as well. Although we observed higher odds ratios (ORs) with regard to LR for CA19‐9 level, pathological stage, and tumor size, there was no statistical significance.

Importantly, for the first time, we present evidence showing that CNI is an independent predictive biomarker for LR in PDAC patients, surpassing the value of LN metastasis status and pathological stage (Table 3). Similarly, plasma CNI has been demonstrated to be a predictor of the response to systemic PDAC therapy and immunotherapy across different types of advanced cancers.^30^, ^31^ More specifically, the CNI might be a prognostic biomarker for PDAC. Herein, we found that a low CNI was significantly correlated with a longer PFS (Figure 3D‐F), which was in accordance with previous data showing that a high CNI was associated with worse clinical outcomes in head and neck squamous cell carcinoma (HNSCC),^32^ gastric cancer,^33^ and PDAC,^34^ respectively. Taken together, lower CNI levels might be a strong signature for the identification of PDAC patients who might benefit from adjuvant therapy after pancreatectomy.

Based on the above findings, we next evaluated the joint utility of CNI and LN metastasis status (Figure 4). Intriguingly, the CNI‐low/LN‐negative subgroup had a particularly higher LR rate than the other three subgroups, indicating that the combination of CNI and LN metastasis status may help identify patients prone to LR after surgery. Genomic analysis is easy to implement with the rapid development of NGS. We have identified a threshold to define CNI‐high and CNI‐low subgroups, and the relationship between CNI and clinical outcome was verified in different NGS panels. Therefore, the joint utility of CNI and LN metastasis status is worth for further investigation and validation in larger size cohorts in the near future.

## 5 CONCLUSION

Taken together, we present evidence demonstrating the association between tumor CNI and clinical outcomes in patients with resected PDAC. Our data indicated that tumor CNI is a potential predictive and prognostic biomarker in patients with resectable PDAC. Notably, the combination of CNI and LN metastasis status may enhance predictive accuracy and improve patient stratification. Further prospective studies are warranted to develop a predictive algorithm to define the optimal threshold for CNI in PDAC and even across different cancer types.

## CONFLICT OF INTEREST

The authors declare that they have no competing interests.

## AUTHOR CONTRIBUTIONS

QZ, BY S, SC, and DC conceived and designed this study. CL W and XX D participated in the coordination and execution of the study. JZ and CH P commented on the study. HC, JC W, JB J, DF C, ZW X, JJ X, WJ Q, and JN G collected the specimens and clinical data. XS, DD R, and CL W analyzed the data and interpreted of results. CL W, XX D, and DD R drafted the manuscript. QZ, BY S, DC, and SC provided critical comments, suggestions, and revised the manuscript. All authors read and approved the final version of the manuscript.

## ETHICS APPROVAL AND CONSENT TO PARTICIPATE

This study was performed in accordance with the ethical standards and the Declaration of Helsinki and according to national and international guidelines. Surgically procured tumor samples from patients were obtained in the Ruijin Hospital, School of medicine, Shanghai Jiao Tong University, the First Affiliated Hospital, Dalian Medical University, and Fujian provincial hospital, Shengli Clinical Medical College of Fujian Medical University with informed patients' consent for research purposes.

## CONSENT FOR PUBLICATION

All authors have reviewed the manuscript and given consent for publication.

## Supporting information

Fig S1

## Data Availability

The datasets used and/or analyzed during the current study are available from the corresponding author on reasonable request.
