# Supplementary figures and images for: Tumor copy number instability is a significant predictor for late recurrence after radical surgery of pancreatic ductal adenocarcinoma
Source: Cancer Med. 2020 Aug 30;9(20):7626–36. doi: 10.1002/cam4.3425 (PMC7571802; doi:10.1002/cam4.3425)

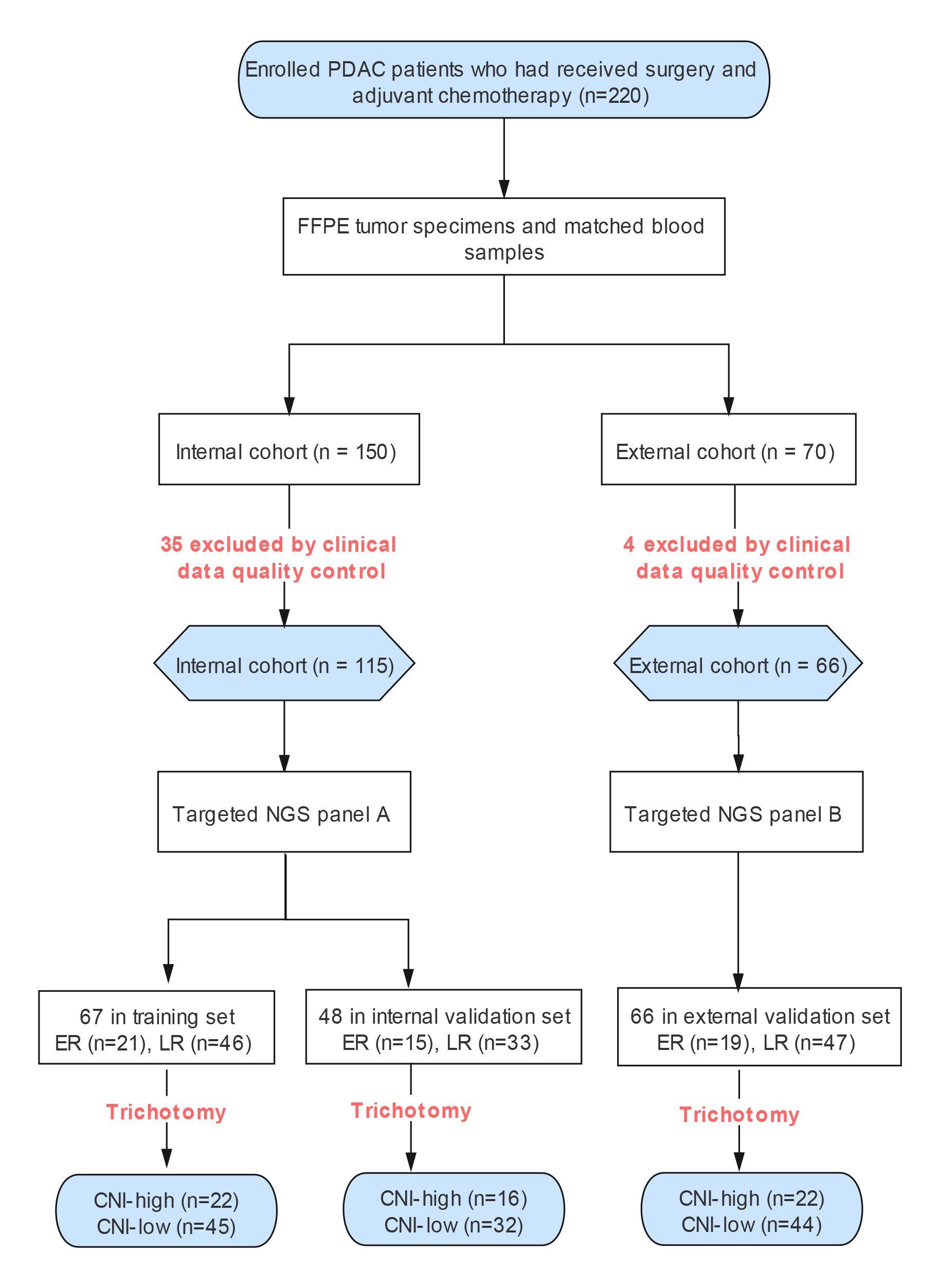

Supplement: Supplementary file 1 — Fig S1 [file CAM4-9-7626-s001.tif]
